# Effects of Spicy Food on Sense of Fairness: Mediating Effects of Aggression and Pathogen Avoidance

**DOI:** 10.1002/pchj.70059

**Published:** 2025-10-07

**Authors:** Weixi Wan, Yanping Shangguan, Qi Wu

**Affiliations:** ^1^ Department of Psychology, School of Educational Science Hunan Normal University Changsha China; ^2^ Cognition and Human Behavior Key Laboratory of Hunan Province Hunan Normal University Changsha China

**Keywords:** aggression, behavioral immune system, pathogen avoidance, sense of fairness, spicy food

## Abstract

Spicy food consumption is prevalent worldwide, yet its psychological and behavioral impacts remain underexplored compared to basic tastes like sweet, sour, salty, bitter, and umami. The present research aimed to investigate the effects of spicy food preferences and consumption on individuals' sense of fairness, with aggression and pathogen avoidance considered as potential mediators. Two behavioral studies using the Ultimatum Game were conducted to examine these relationships. Study 1 found that individuals with a preference for spicy food were more likely to reject unfair offers and accept fair offers, mediated by trait aggression and trait pathogen avoidance, respectively. In Study 2, immediate consumption of spicy food led to a higher rejection rate of unfair offers compared to non‐spicy food consumption, an effect mediated by increased state aggression. However, no significant differences in fair offer rejection rates were observed between the spicy and non‐spicy conditions, and no significant mediation effects of situational pathogen avoidance were detected. These findings suggest that spicy food enhances sensitivity to unfairness—likely by elevating an individual's acceptance threshold—an effect primarily driven by aggression. This research provides novel insights into how sensory experiences shape social decision‐making and fairness judgments.

## 1 Introduction

Throughout evolutionary history, humans as omnivores have faced numerous adaptive challenges from diseases, parasites, toxins, and predators, leading to the development of critical abilities to distinguish between toxic and edible foods—abilities crucial for both immediate survival and long‐term health and well‐being (Tooby and DeVore 1987). This evolutionary imperative shaped human gustatory preferences, making food selection a fundamental aspect of survival that supports various adaptive systems (e.g., Krebs 2009; Spence 2018; Tooby and DeVore 1987). Given the profound influence of taste preferences on human psychology and behavior (Chen and Chang 2012; Meier et al. 2012; Sagioglou and Greitemeyer 2014; Vi and Obrist 2018; Zhang et al. 2023), the present research investigates a largely unexplored question: how do preferences for and consumption of spicy foods influence individuals' sense of fairness in social interactions? Specifically, we examine whether spicy food affects fairness‐related decision‐making through two key psychological mechanisms—aggression and pathogen avoidance—that have evolutionary significance for human cooperation and survival.

Research demonstrates that evolutionarily shaped dietary preferences are linked to distinct psychological processes and personality traits. People typically prefer sweet and salty foods for their biological benefits while avoiding bitter and sour foods that signal potential harm (Krebs 2009; Meier et al. 2012; Sagioglou and Greitemeyer 2014). These preferences extend beyond nutrition to influence psychology: sweet food preferences correlate with agreeableness and prosociality, while bitter preferences associate with antisocial traits (Meier et al. 2012; Sagioglou and Greitemeyer 2014). Gustatory experiences also affect emotional responses and social decision‐making, with sweet flavors enhancing positive emotions and prosocial behaviors, bitter flavors evoking negative emotions and hostile intentions, and sour flavors prompting adventurous behaviors and feelings of envy (Chen and Chang 2012; Vi and Obrist 2018; Zhang et al. 2023). These patterns suggest that spicy food preferences, like other taste preferences, may have important implications for social decision‐making and behavior.

Spicy food preferences present a particularly intriguing case. Unlike the clear survival benefits of sweet foods (energy) or the obvious dangers of bitter foods (toxins), spicy foods involve voluntary consumption of pain‐inducing substances (e.g., Byrnes and Hayes 2016; Spence 2018). Yet research demonstrates distinct human preferences for spicy flavors, especially in regions with high pathogen exposure (Bromham et al. 2021; Chen et al. 2024; Prokop and Fančovičová 2011; Spence 2018). The primary source of spiciness in many cuisines worldwide comes from chili peppers, which possess potent antibacterial and antimicrobial properties, neutralizing harmful bacteria such as *Bacillus*, *Streptococcus*, and *Staphylococcus* (Spence 2018). This suggests that spicy food consumption may have evolved as an adaptive response to environmental challenges, offering protective benefits beyond culinary pleasure (Chen et al. 2024; Prokop and Fančovičová 2011; Spence 2018). However, the psychological and behavioral consequences of these spicy food preferences remain underexplored, particularly regarding their potential influence on social cooperation and fairness—fundamental aspects of human social life.

### 1.1 Spicy Food, Aggression, and Sense of Fairness

The first potential mechanism linking spicy food consumption to the sense of fairness involves aggression. Research reveals robust connections between spicy food consumption and aggressive tendencies operating at both trait and state levels. At the trait level, individuals with preferences for spicy foods show correlations with sensation‐seeking, risk‐taking, irritability, and aggressive behaviors (Batra et al. 2017; Bègue et al. 2015; Chen et al. 2024; Ji et al. 2013; Ludy and Mattes 2012). These associations appear rooted in biological mechanisms: individuals with higher testosterone levels display a greater inclination toward consuming spicy food, while elevated testosterone is associated with increased aggression (Bègue et al. 2015; Burnham 2007).

Beyond trait‐level associations, consuming spicy foods directly influences aggressive state through multiple mechanisms. The consumption leads to increased excitement through heightened secretion of neurotransmitters like norepinephrine, dopamine, and endorphins (Byrnes and Hayes 2016). The experience of consuming spicy foods, characterized by burning sensations, can trigger aggressive tendencies through embodied cognition, while neuroendocrine responses including testosterone release and accompanying discomfort can exacerbate aggression (Batra et al. 2017; Bègue et al. 2015; Byrnes and Hayes 2016). Research demonstrates that spicy food consumption augments individuals' state aggression and facilitates perception of angry expressions (Batra et al. 2017; Chen et al. 2024).

These spicy food–aggression links become particularly relevant when considering how aggression influences fairness‐related decision‐making. Cooperation and fairness assessment are fundamental to human survival and social functioning (Jost and Kay 2010). Research consistently demonstrates that individual aggressiveness significantly influences the sense of fairness through heightened sensitivity to threatening information and hostile attribution to perceived threats (Evans et al. 2024). Aggressive individuals are more likely to reject unfair offers by perceiving them as aggressive behaviors during cooperation and experience more anger when confronted with unfair proposals—anger that frequently manifests in the rejection of unfair offers (Evans et al. 2024; Rubio‐Garay et al. 2016; Seip et al. 2014; Zheng et al. 2017). This suggests that individuals who prefer or consume spicy foods may exhibit an altered sense of fairness through aggression‐mediated pathways, potentially leading to higher rejection rates of unfair offers.

### 1.2 Spicy Food, Behavioral Immune System, and Sense of Fairness

The second potential mechanism involves the behavioral immune system and pathogen avoidance. Humans possess a behavioral immune system that identifies potential pathogens threatening health and survival, activating cognitive and emotional responses that lead to disease avoidance behaviors (Ackerman et al. 2021; Batres and Perrett 2020; Liuzza et al. 2019). This system's activation is typically accompanied by feelings of disgust, which serves as its core component (Culpepper et al. 2018; Murray and Schaller 2016). The behavioral immune system follows a “smoke detector” principle, tending to perceive anything that deviates from norms as pathogen cues and eliciting disgust (Ackerman et al. 2018; Wormley and Varnum 2023).

This pathogen detection system extends beyond physical threats to social violations. Research suggests that the behavioral immune system perceives violations of fairness norms as pathogen cues, subsequently leading to increased rejection and avoidance of perpetrators (Hlay et al. 2021). Studies on moral judgment and disease avoidance indicate that disgust toward immoral actions serves as disease prevention, with individuals associating norm violators with pathogens—an association that becomes stronger when disease threats are salient, leading to exclusion and punishment intentions (Brenner and Inbar 2015). Activating the behavioral immune system increases individuals' disgust toward violations of social norms and promotes conformity to morality (Schwambergová et al. 2023; Wu and Chang 2012).

The connection between disgust and fairness decisions is well‐established. Individuals with high disgust sensitivity tend to be more stringent in moral judgments (Brenner and Inbar 2015). Research confirms that disgust prompts more rejections than other emotions in the Ultimatum Game, and both experimentally induced disgust and exposure to disgusted expressions lead to a higher likelihood of rejecting unfair proposals (Harlé and Sanfey 2010; Liu et al. 2016).

Linking these findings to spicy food, given the disease‐preventive properties of spicy foods discussed above, existing studies indicate that individuals preferring spicy food exhibit higher trait activation levels of the behavioral immune system (trait pathogen avoidance; Prokop and Fančovičová 2011; Spence 2018). Recent research found that the preference for spicy food positively correlates with the perceptual accuracy of disgusted facial expressions, mediated by trait pathogen avoidance (Chen et al. 2024). These findings suggest that individuals favoring spicy food have higher trait pathogen avoidance, potentially making them more sensitive to norm violations and more likely to reject unfair offers as pathogen‐related threats.

However, the relationship between immediate spicy food consumption and situational pathogen avoidance presents a more complex picture. While spicy food consumption can theoretically be interpreted as fulfilling pathogen avoidance needs due to chili peppers' antibacterial properties, prior research found that eating spicy foods has no immediate effect on situational pathogen avoidance (Chen et al. 2024). This suggests that while trait‐level pathogen avoidance may mediate relationships between spicy preferences and fairness, immediate consumption effects may operate through different mechanisms.

### 1.3 The Current Research

Building on these two theoretical mechanisms, we conducted two complementary studies to provide the first empirical examination of how spicy food influences the sense of fairness. Our research addresses this gap by investigating both trait‐level preferences and state‐level consumption effects, testing whether the established connections between spicy food and aggression, as well as spicy food and pathogen avoidance, translate into meaningful differences in fairness behavior.

Study 1 employed a correlational design to examine trait‐level relationships between individuals' spicy food preferences and their sense of fairness, measured through rejection rates in the Ultimatum Game (hereafter abbreviated as UG; Shang et al. 2025; Wu et al. 2013). Based on the literature linking spicy preferences to aggressive traits (e.g., Batra et al. 2017; Bègue et al. 2015; Ji et al. 2013; Ludy and Mattes 2012) and pathogen avoidance tendencies (e.g., Chen et al. 2024; Prokop and Fančovičová 2011; Spence 2018), we hypothesized that:

*Stronger preference for spicy food would be associated with higher rejection rates to unfair offers*.

*The relationship between spicy food preference and unfair offer rejection would be mediated by higher trait aggression*.

*The relationship between spicy food preference and unfair offer rejection would be mediated by higher trait pathogen avoidance*.

Study 2 employed an experimental design to examine state‐level causal effects of spicy food consumption on individuals' sense of fairness. Based on research demonstrating that consuming spicy foods can temporarily increase aggressive states, we hypothesized that:

*Participants consuming spicy food would reject more unfair offers compared to a control group*.

*The effect of spicy food consumption on unfair offer rejection would be mediated by increased state aggression*.

Given the limited and mixed evidence regarding immediate effects of spicy food consumption on pathogen avoidance states (Chen et al. 2024), we explored but did not make a definitive prediction about mediation through situational pathogen avoidance in Study 2. Through these investigations, we aimed to provide an initial examination of how sensory experiences, particularly spicy taste, shape social decision‐making and fairness judgments.

## 2 Study 1

### 2.1 Methods

#### 2.1.1 Participants

Utilizing G*Power software, a priori estimate of the required sample size for this study showed that the adequate sample size for an anticipated “medium effect” (*f* ^2^ = 0.15; e.g., Chen et al. 2024; Cooper and Findley 1982; Kempthorne and Terrizzi Jr. 2021; Wu et al. 2022; Yang et al. 2025) would be at least *n* = 99 in Study 1 (*α* = 0.05, power = 0.90). Finally, we recruited 105 Chinese college students (49 males, 56 females, *M*_age_ = 20.42, SD = 2.45) from the authors' institutions to take part in this study, with no history of spicy food allergy. All participants had normal visual acuity or corrected visual acuity, and had not joined similar studies. All participants provided their written informed consent in accordance with the Declaration of Helsinki.

#### 2.1.2 Spicy Food Preference

Spicy food preference was measured in the same way as Batra et al. (2017) and Chen et al. (2024). Participants were asked to rate their preference for spicy food (“How spicy is the food you typically consume?”) on a scale of 1–100 (1 = “*not at all spicy*”, 100 = “*very spicy*”). This single‐item approach has been widely employed in previous studies examining spicy food preferences (e.g., Bègue et al. 2015; Chen et al. 2024; Ji et al. 2013; Prokop and Fančovičová 2011).

#### 2.1.3 Trait Aggression

Buss‐Perry Aggression Questionnaire was adopted to measure the trait aggression of the participants (BP‐AQ, Chinese‐translated version, Li et al. 2011). The questionnaire consists of five subscales: physical aggression, verbal aggression, anger, hostility, and indirect aggression. Consistent with prior research (e.g., Chen et al. 2024), we used the total score of the five subscales as a single indicator of trait aggression (Cronbach's *α* = 0.89). The scales were scored on a 5‐point scale ranging from 1 (*not compliant*) to 5 (*fully compliant*), and the higher scores indicated higher trait aggression.

#### 2.1.4 Trait Pathogen Avoidance

The Perceived Vulnerability to Disease (PVD) Scale was utilized to evaluate participants' trait pathogen avoidance (e.g., Chen et al. 2024; Duncan et al. 2009; Wu and Chang 2012; Wu et al. 2015, 2022). The scale consists of 15 items divided into two subscales, Germ Aversion (GA; e.g., “My hands do not feel dirty after touching money”) and Perceived Infectability (PI; e.g., “If illness is going around, I will get it”). Participants rated on a 7‐point scale, ranging from 1 (*strongly disagree*) to 7 (*strongly agree*). Six items were reverse scored. In the present sample, the internal consistency of the GA subscale was low (Cronbach's *α* = 0.55), while the PI subscale demonstrated acceptable reliability (Cronbach's *α* = 0.76). Some previous research has also reported relatively lower reliability for the GA subscale (Cronbach's *α* = 0.55–0.61; e.g., Chen et al. 2024; Wu and Chang 2012).

Due to the widespread use of the total PVD score as an indicator of trait pathogen avoidance in behavioral immune system research, and given that previous studies have consistently shown acceptable internal consistency for the total PVD score (Cronbach's *α* > 0.7; e.g., Duncan et al. 2009; Wu and Chang 2012; Wu et al. 2015), we used the total scores of the PVD scale as the indicator of participants' trait pathogen avoidance in Study 1 (Cronbach's *α* = 0.68, composite reliability *ρ*_Y_ = 0.75^1^). Higher scores indicate higher trait pathogen avoidance.

#### 2.1.5 UG Task

Study 1 employed the UG task (e.g., Shang et al. 2025; Wu et al. 2013) to measure participants' sense of fairness through their acceptance/rejection choices. Participants were informed that they would engage in a real money‐distributing game over the Internet with three other players. These three opponents, designated as allocators, were responsible for dividing a sum of 100 yuan provided for the study, with participants' final payment based on their average earnings across trials. Prior to the formal task, the experimenter confirmed that all participants understood the payment procedure to ensure task credibility. In each trial, participants would randomly interact with one of these three opponents and were instructed to press “F” or “J” on the keyboard to either reject or accept the allocation (key assignments randomly balanced). Rejecting the allocation would result in no monetary gain for either player, whereas accepting it would result in settlement based on the opponent's distribution scheme.

**FIGURE 1:**
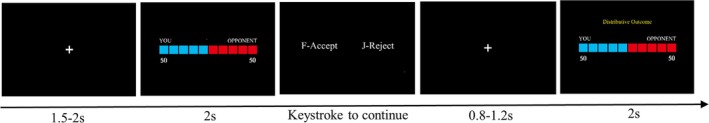
UG task. The original experiment was conducted in Chinese.

Each participant first completed 4 practice trials before proceeding to the formal task, which consisted of 60 trials: 30 fair allocations (50:50) and 30 unfair allocations (70:30, 80:20, and 90:10, with 10 instances each). Each trial started with a fixation cross appearing for 1.5–2 s (See Figure 1). Subsequently, participants were shown the opponent's distribution scheme for 2 s, depicted by two colored bars (blue = participant's amount, red = opponent's amount). Participants made their decision with no time constraint until the screen disappeared following their choice. Upon accepting or rejecting the offer via keystroke, another fixation cross appeared for 0.8–1.2 s, followed by their earnings for the current trial displayed for 2 s. The payoffs for each round were recorded, and average payoffs served as final experimental compensation.

#### 2.1.6 Procedure

Participants completed the spicy food preference rating, trait measures (BP‐AQ and PVD), and UG task, presented as separate unrelated studies.

#### 2.1.7 Data Analysis

Study 1 employed a correlational design to examine relationships between spicy food preferences and sense of fairness. To test H1a, we first conducted a one‐way repeated‐measures analysis of variance (ANOVA) with allocation type (fair vs. unfair) as the within‐subjects factor to confirm the basic UG effect. We then employed linear regression analyses to examine how spicy food preference predicted rejection rates for unfair and fair offers separately. We also conducted detailed linear regression analyses examining spicy food preference effects on each specific unfairness level (70:30, 80:20, 90:10) to further examine the effects across different inequality levels. All variables were standardized prior to regression analyses.

To test H1b and H1c, we conducted mediation analyses using the SPSS PROCESS macro (Model 4; Hayes and Preacher 2014) with bootstrap estimation (5000 samples, 95% bias‐corrected confidence intervals). We tested separate mediation models for unfair and fair offers, with spicy food preference as a predictor and trait aggression and trait pathogen avoidance as simultaneous mediators.

### 2.2 Results

The repeated‐measures ANOVA confirmed the basic UG effect: participants rejected unfair offers significantly more than fair offers, *F* (1, 104) = 445.95, *p* < 0.001, *η*_p_^2^ = 0.85, 95% CI [57.71, 66.99], with unfair rejection rates substantially exceeding fair rejection rates. Descriptive statistics and zero‐order correlations are presented in Table 1.

**TABLE 1:** Descriptive statistics for the variables and the correlation coefficient matrix (Study 1).

	1	2	3	4	5	6	7	8
1. Spicy food preference	1							
2. Trait pathogen avoidance	0.19	1						
3. Trait aggression	0.47**	0.17	1					
4. Unfair (overall)	0.57**	0.11	0.44***	1				
5. Fair: 50:50	−0.06	−0.26**	−0.14	0.04	1			
6. Unfair: 70:30	0.400***	0.08	0.21*	0.75***	0.20*	1		
7. Unfair: 80:20	0.401***	0.09	0.30**	0.76***	0.12	0.60***	1	
8. Unfair: 90:10	0.22*	0.01	0.31***	0.56***	−0.01	0.29**	0.70***	1
*M*	57.09	58.66	66.83	68.51	6.16	47.62	72.38	82.67
SD	19.85	9.49	14.14	22.51	9.20	36.91	31.85	24.86

*Note*: Rejection rates are presented as percentages. *p ≤ 0.05; **p ≤ 0.01; ***p ≤ 0.001.

Further regression analyses examined how spicy food preference predicted fairness decisions. Spicy food preference positively predicted unfair offer rejection rates (*β* = 0.57, SE = 0.08, *t* (103) = 7.03, *p* < 0.001, 95% CI [0.41, 0.73]), supporting H1a. Spicy food preference did not significantly predict fair offer rejection rates (*β* = −0.06, SE = 0.10, *t* (103) = −0.62, *p* = 0.534, 95% CI [−0.26, 0.13]).

Detailed analyses for specific unfairness levels showed that spicy food preference positively predicted rejection rates for 70:30 offers (*β* = 0.400, SE = 0.09, *t* (103) = 4.43, *p* < 0.001, 95% CI [0.221, 0.579]), 80:20 offers (*β* = 0.401, SE = 0.09, *t* (103) = 4.44, *p* < 0.001, 95% CI [0.221, 0.580]), and 90:10 offers (*β* = 0.22, SE = 0.10, *t* (103) = 2.28, *p* < 0.001, 95% CI [0.03, 0.41]). These consistent effects across unfairness levels, especially the nearly identical effects for 70:30 and 80:20 offers, suggested spicy food preference increased participants' acceptance threshold and enhanced their sensitivity to all levels of unfairness. Given these consistent effects and significant intercorrelations among rejection rates for different unfairness levels (see Table 1), we used the mean unfair offer rejection rate for mediation analyses.

The mediation analyses showed that spicy food preference positively predicted trait aggression (*β* = 0.47, SE = 0.09, *t* (103) = 5.45, *p* < 0.001, 95% CI [0.30, 0.65]) and marginally predicted trait pathogen avoidance (*β* = 0.19, SE = 0.10, *t* (103) = 1.94, *p* = 0.055, 95% CI [−0.004, 0.38]). As for unfair offers, trait aggression significantly predicted rejection rates (*β* = 0.22, SE = 0.09, *t* (101) = 2.37, *p* = 0.019, 95% CI [0.04, 0.40]), while trait pathogen avoidance did not (*β* = −0.01, *p* = 0.867). The indirect effect through trait aggression was significant (*β* = 0.10, SE = 0.05, 95% CI [0.01, 0.20]), supporting H1b, while the indirect effect through trait pathogen avoidance was not significant (*β* = −0.003, SE = 0.02, 95% CI [−0.04, 0.03]), failing to support H1c.

**FIGURE 2:**
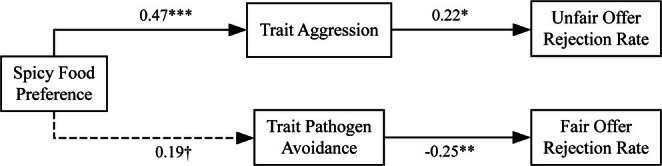
Significant mediation effects found in Study 1. ^†^ *p* ≤ 0.06, *p ≤ 0.05; **p ≤ 0.01, ***p ≤ 0.001.

For fair offers, trait aggression did not significantly predict rejection rates (*β* = −0.12, *p* = 0.289), while trait pathogen avoidance negatively predicted rejection rates (*β* = −0.25, SE = 0.10, *t* (101) = −2.56, *p* = 0.012, 95% CI [−0.44, −0.06]). The indirect effect through trait aggression was not significant (*β* = −0.06, SE = 0.05, 95% CI [−0.16, 0.05]), while the indirect effect through trait pathogen avoidance was significant (*β* = −0.05, SE = 0.03, 95% CI [−0.014, −0.002]) (see Figure 2).

### 2.3 Discussion

Study 1 provided support for H1a and H1b but not H1c. As hypothesized (H1a), stronger preferences for spicy food were associated with heightened rejection of unfair offers. This relationship was significantly mediated by trait aggression (H1b), consistent with previous research linking spicy food preferences to aggressive traits (Batra et al. 2017; Bègue et al. 2015; Chen et al. 2024). However, trait pathogen avoidance did not mediate the relationship between spicy food preference and unfair offer rejection (H1c).

Additionally, the results revealed that trait pathogen avoidance significantly mediated the relationship between spicy food preference and fair offer acceptance. Individuals with stronger spicy food preferences exhibited higher trait pathogen avoidance, which predicted greater acceptance of fair distributions. This finding aligns with behavioral immune system theory, where norm compliance serves as a threat management strategy (Ackerman et al. 2018).

The analysis across different unfairness levels revealed consistent positive effects of spicy food preference on rejection rates, with particularly similar effects for 70:30 and 80:20 offers, suggesting that spicy food preference increased individuals' acceptance threshold and enhanced their sensitivity to unfairness across different inequality levels. These correlational findings provide initial evidence that trait‐level spicy food preferences influence individuals' sense of fairness through aggression‐related mechanisms. However, to establish causal relationships regarding the immediate state‐level effects of spicy food consumption on fairness decisions, experimental investigation was necessary. Study 2 therefore employed an experimental design to test whether consuming spicy foods acutely affects sense of fairness and whether similar mechanisms (aggression and pathogen avoidance) mediate these state‐level effects.

## 3 Study 2

### 3.1 Methods

#### 3.1.1 Participants

By utilizing G*Power, a priori estimate of the required sample size showed that the adequate sample size for Study 2 (*f* ^2^ = 0.15, power = 0.90, *α* = 0.05; e.g., Chen et al. 2024; Cooper and Findley 1982; Kempthorne and Terrizzi Jr. 2021; Wu et al. 2022; Yang et al. 2025) would be at least *n* = 130. Finally, we recruited 132 Chinese college students (37 males, 93 females, *M*_age_ = 19.38, SD = 2.25) from authors' institutions to take part in this study, with no history of spicy food allergy. All participants had normal visual acuity or corrected visual acuity and had not joined in similar experiments. All participants provided their written informed consent in accordance with the Declaration of Helsinki.

#### 3.1.2 State Aggression

We employed an ambiguous aggressive behavior judgment task to gauge the participants' state aggression (Batra et al. 2017; Chen et al. 2024). During this part, participants were presented with a vignette featuring a protagonist named “Jay” engaging in behavior that was deliberately vague in terms of aggression. Subsequently, they were asked to rate the degree to which “Jay” demonstrated aggressive intentions through the following three statements, with which participants indicated their degree of agreement on a scale ranging from 1 (*completely disagree*) to 7 (*completely agree*): “Jay” (the protagonist) is “hot‐headed”, “short‐tempered,” and “aggressive” (Cronbach's *α* = 0.63, composite reliability *ρ*_Y_ = 0.81). To obscure the true objective of the task, participants were also required to rate “Jay” on two additional unrelated personality traits: “assertive” and “impulsive.”

#### 3.1.3 Situational Pathogen Avoidance

Study 2 utilized the Culpepper Disgust Image Set (C‐DIS) to assess participants' situational pathogen avoidance (Chen et al. 2024; Culpepper et al. 2018). The image set comprises 20 pathogen salient images (e.g., a dirty unflushed toilet) and 20 visually similar control images devoid of any pathogen disgust elicitors. Participants were instructed to evaluate each image on a scale ranging from 0 (*not disgusting at all*) to 6 (*very disgusting*). We calculated the total disparity between the disgust ratings of the pathogen salient images and those of the control images as the indicator of participants' situational pathogen avoidance (*α* = 0.95; Chen et al. 2024; Culpepper et al. 2018).

#### 3.1.4 Manipulation of Spicy Food Consumption

The identical procedure and food of Chen et al. (2024) was employed to manipulate the spicy food consumption in Study 2. Specifically, participants were instructed to eat either spicy food (consisting of 25 g of dried bean curd mixed with 2.5 g of chili pepper) or non‐spicy food (solely the 25 g of dried bean curd). This manipulation has been demonstrated to be effective, showing that the spicy food was perceived as significantly spicier than the non‐spicy food and that the overall aversion towards the food had no significant difference between participants in the two conditions (Chen et al. 2024). Furthermore, the study of Chen et al. (2024) also demonstrated that no sensory adaptation phenomenon was observed in participants when they washed up after this manipulation, rested for 5 min, and then employed this manipulation again. Participants experienced the same feeling as they did the first time (Chen et al. 2024).

#### 3.1.5 Procedure

Participants were first asked to sign an informed consent and were told that they needed to complete three unrelated tasks. The participants were then randomly assigned to the spicy food condition or the non‐spicy food condition. Then participants were asked to eat a certain amount of dried beans, with the participants in the spicy food condition eating dried beans with a spicy flavor and those in the non‐spicy food condition eating plain dried beans without a spicy flavor. After eating the offered food, the participants were instructed to rate their current mood state on a scale of 0 (*very happy*) to 8 (*very unhappy*) and their degree of emotional arousal on a scale of 0 (*very calm*) to 8 (*very excited*). Subsequently, the participants completed the UG task (same as Study 1). The UG task was administered immediately after spicy food consumption considering that the immediate effects of spicy food might be limited in duration (e.g., Batra et al. 2017; Chen et al. 2024). Additionally, since the C‐DIS contains salient pathogen threat images that could potentially alter participants' responses to unfairness (given previous research demonstrating disgust effects on fairness judgments; e.g., Harlé and Sanfey 2010; Liu et al. 2016), we administered the UG task before exposure to these images to avoid confounding effects. Following the UG task, the participants rinsed their mouths with purified water and took a 10‐min break to ensure that there was no spicy taste in their mouths. At the end of the break, the participants ate an equal amount of dried beans with the same flavor as before and then completed the ambiguous aggressive behavior judgment task and rated the C‐DIS images, with the order of completion of the two tasks being randomized.

#### 3.1.6 Data Analysis

Study 2 employed an experimental design to examine causal effects of spicy food consumption on sense of fairness. To test H2a, we conducted a 2 (spicy food consumption condition: spicy vs. non‐spicy) × 2 (allocation type: fair vs. unfair) mixed‐model ANOVA with spicy food consumption condition as the between‐subjects factor and allocation type as the within‐subjects factor. We also conducted a detailed 2 × 3 mixed‐design ANOVA examining spicy food consumption effects across different unfairness levels, with spicy food consumption condition (spicy vs. non‐spicy) as the between‐subjects factor and unfairness level (70:30, 80:20, 90:10) as the within‐subjects factor to further examine the effects across different inequality levels. Greenhouse–Geisser corrections were applied when sphericity assumptions were violated.

To test H2b and explore potential mediation through situational pathogen avoidance, we conducted mediation analyses using the SPSS PROCESS macro (Model 4; Hayes and Preacher 2014) with bootstrap estimation (5000 samples, 95% bias‐corrected confidence intervals). We tested separate mediation models for unfair and fair offers, with spicy food consumption condition as predictor, state aggression and situational pathogen avoidance as primary mediators of theoretical interest. Mood state and emotional arousal were included as parallel mediators to provide conservative tests of the aggression and pathogen avoidance pathways, given their documented covariation with both spicy food consumption and disgust responses (Chen et al. 2024; Wu and Chang 2012; Wu et al. 2022; Yang et al. 2025). All variables were standardized prior to analyses.

### 3.2 Results

The 2 × 2 mixed‐model ANOVA revealed a significant main effect of allocation type, with participants rejecting unfair offers significantly more than fair offers (see Table 2), *F* (1, 130) = 1011.00, *p* < 0.001, *η*_p_^*2*^ = 0.89, 95% CI [54.85, 65.44], supporting the basic UG effect. A significant main effect of spicy food consumption condition was also found, *F* (1, 130) = 10.56, *p* < 0.001, *η*_p_^*2*^ = 0.08, 95% CI [2.12, 12.41], with participants in the spicy food condition showing higher overall rejection rates than those in the non‐spicy food condition (see Table 2). The interaction between spicy food consumption condition and allocation type was significant, *F* (1, 130) = 12.02, *p* = 0.001, *η*_p_^2^ = 0.085.

**TABLE 2:** Descriptive statistics for all measured variables by condition in Study 2.

Variables	Spicy food		Non‐spicy food	
	*M*	SD	*M*	SD
Rejection rate (%)				
Unfair offers (Overall)	72.11	22.37	58.28	22.97
70:30 offer	46.67	39.70	32.12	30.21
80:20 offer	77.88	29.01	65.00	33.02
90:10 offer	91.97	13.50	81.36	23.20
Fair offers (50:50)	5.40	7.78	4.70	6.61
Mediating and control variables				
State aggression	15.34	2.77	14.18	3.02
Situational pathogen avoidance	62.57	17.25	64.12	17.60
Mood	4.74	1.26	5.18	1.25
Emotional arousal	5.18	1.64	4.56	1.40

*Note*: Rejection rates are presented as percentages.

Simple effects analyses revealed that participants in the spicy food condition rejected unfair offers significantly more than those in the non‐spicy food condition (see Table 2), *F* (1, 130) = 12.27, *p* < 0.001, *η*_p_^*2*^ = 0.09, 95% CI [6.02, 21.63], supporting H2a. However, no significant difference was found for fair offer rejection rates between the spicy food condition and the non‐spicy food condition, *F* (1, 130) = 0.32, *p* = 0.573, *η*_p_^2^ = 0.002.

The detailed 2 × 3 mixed‐design ANOVA examining spicy food consumption effects across different unfairness levels revealed a significant main effect of unfairness level, *F* (1.72, 260) = 149.54, *p* < 0.001, *η*_p_^2^ = 0.54, and a significant main effect of spicy food consumption condition, *F* (1, 130) = 10.31, *p* = 0.002, *η*_p_^2^ = 0.07. However, the interaction between spicy food consumption condition and unfairness level was not significant (*F* (1.72, 260) = 0.25, *p* = 0.778, *η*_p_^2^ = 0.002), suggesting that spicy food consumption increased participants' acceptance threshold and enhanced their sensitivity to unfairness across all levels. Given these consistent effects across unfairness levels and following the analytical approach from Study 1, we used the mean unfair offer rejection rate for mediation analyses.

**FIGURE 3:**
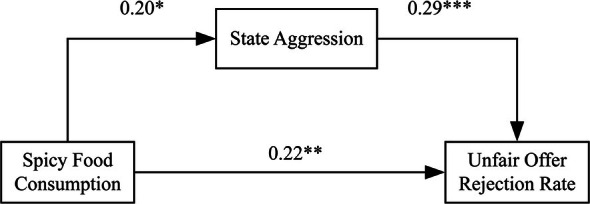
Significant mediation effect found in Study 2. *p ≤ 0.05; **p ≤ 0.01; ***p ≤ 0.001.

Subsequent mediation analyses showed that spicy food consumption significantly increased participants' state aggression (*β* = 0.20, SE = 0.09, *t* (130) = 2.31, *p* = 0.022, 95% CI [0.03, 0.37]), but had no significant effect on situational pathogen avoidance (*β* = −0.04, SE = 0.09, *t* (130) = −0.51, *p* = 0.611, 95% CI [−0.22, 0.13]; see Figure 3). Spicy food consumption also significantly affected mood state (*β* = −0.35, SE = 0.08, *t* (130) = −4.01, *p* < 0.001, 95% CI = [−0.52, −0.18]) and emotional arousal (*β* = 0.20, SE = 0.09, *t* (130) = 2.34, *p* = 0.02, 95% CI [0.03, 0.37]).

For unfair offers, among the primary mediators, state aggression significantly predicted rejection rates (*β* = 0.29, SE = 0.08, *t* (126) = 3.38, *p* = 0.001, 95% CI [0.12, 0.45]), while situational pathogen avoidance did not (*β* = 0.02, *p* = 0.82). The mediation analysis revealed that state aggression significantly mediated the effect of spicy food consumption on unfair offer rejection rates (*β* = 0.06, SE = 0.03, 95% CI [0.01, 0.12]), supporting H2b. The indirect effect through situational pathogen avoidance was not significant (*β* = −0.001, SE = 0.01, 95% CI [−0.02, 0.02]). The indirect effects through mood state (*β* = 0.02, SE = 0.02, 95% CI [−0.02, 0.06]) and emotional arousal (*β* = 0.0002, SE = 0.02, 95% CI [−0.05, 0.04]) were also not significant (see Figure 3).

For fair offers, neither state aggression (*β* = 0.09, SE = 0.09, *t* (126) = 1.02, *p* = 0.311, 95% CI [−0.09, 0.28]) nor situational pathogen avoidance (*β* = −0.07, SE = 0.09, *t* (126) = −0.79, *p* = 0.432, 95% CI [−0.25, 0.11]) significantly predicted rejection rates. Accordingly, no significant indirect effects were found for the primary mediators: state aggression (*β* = 0.02, SE = 0.02, 95% CI [−0.02, 0.07]) and situational pathogen avoidance (*β* = 0.003, SE = 0.01, 95% CI [−0.02, 0.03]). The indirect effects of mood state (*β* = 0.01, SE = 0.02, 95% CI [−0.04, 0.06]) and emotional arousal (*β* = 0.01, SE = 0.02, 95% CI [−0.02, 0.07]) were also not significant.

### 3.3 Discussion

Study 2 provided experimental evidence for the causal effects of spicy food consumption on the sense of fairness. Supporting H2a, participants who consumed spicy food demonstrated significantly higher rejection rates for unfair offers compared to those in the control condition, establishing that immediate spicy food consumption produces state‐level effects on individuals' sense of fairness. The effect remained consistent across all levels of unfairness, indicating that spicy food consumption elevated participants' acceptance threshold and enhanced their sensitivity to unfairness across different degrees of inequality.

The mediation analysis further examined the psychological pathways underlying this effect and supported H2b. Results revealed that increased state aggression significantly mediated the relationship between spicy food consumption and unfair offer rejection. The experimental manipulation successfully increased participants' state aggression, which in turn heightened their sensitivity to unfairness and increased rejection of inequitable offers. This confirms that aggression serves as a key psychological mechanism through which immediate spicy food consumption influences the sense of fairness.

In contrast, situational pathogen avoidance did not serve as a mediating pathway. The spicy food manipulation did not significantly affect participants' situational pathogen avoidance responses, and this variable did not predict fairness behavior in the experimental context, suggesting that the behavioral immune system pathway may not be influenced by immediate spicy food consumption.

## 4 General Discussion

The present research provides the first empirical investigation into the relationship between spicy food and the human sense of fairness. Across two complementary studies, we identified a consistent and robust pattern: both a dispositional preference for and the immediate consumption of spicy food are linked to a reduced tolerance for unfairness in social exchanges. This manifested as an elevated acceptance threshold, reflecting a heightened sensitivity to inequity that was applied consistently across all unfair offers. Critically, this effect was consistently mediated by aggression at both the trait (Study 1) and state (Study 2) levels. In contrast, the role of pathogen avoidance was more complex and did not emerge as a significant pathway influencing responses to unfairness. These findings extend our understanding of how fundamental sensory experiences, shaped by evolutionary pressures, can profoundly influence sophisticated social decision‐making.

### 4.1 The Robust Link Among Spicy Food, Aggression, and a Heightened Sense of Fairness

Our findings provide converging evidence for a powerful psychological pathway connecting spicy food, aggression, and fairness judgments. In Study 1, we established that individuals with a stronger preference for spicy food were more likely to reject unfair offers (supporting H1a), a relationship significantly accounted for by their higher levels of trait aggression (supporting H1b). Study 2 provided causal support for this link, demonstrating that the acute consumption of spicy food increased participants' rejection rate of unfair proposals (supporting H2a), an effect that was in turn mediated by an increase in state aggression (supporting H2b).

These results align with and extend a growing body of literature. Previous research has consistently documented a positive association between spicy food preference and aggressive tendencies (Batra et al. 2017; Bègue et al. 2015; Chen et al. 2024; Ji et al. 2013), as well as the capacity of spicy food consumption to induce an aggressive state and facilitate the perception of anger in others (Batra et al. 2017; Chen et al. 2024). Our research integrates these findings into the domain of social decision‐making, suggesting that spicy food modulates social cognition by elevating aggression. This heightened aggressive state appears to increase an individual's sensitivity to social provocations, leading them to interpret unfair offers not merely as inequitable but as direct threats or challenges (e.g., Bègue et al. 2015; Burnham 2007). In this context, the rejection of an unfair offer can be understood as a form of reactive aggression—a non‐physical but potent response to perceived social slights and status threats (Carré et al. 2011).

### 4.2 Spicy Food and the Nuanced Role of Pathogen Avoidance

In contrast to the clear role of aggression, the link between spicy food, pathogen avoidance, and fairness was more nuanced. Our hypothesis that trait pathogen avoidance would mediate the relationship between spicy food preference and the rejection of unfair offers (H1c) was not supported in Study 1. Similarly, in Study 2, where we explored this pathway at the state level, the consumption of spicy food did not influence state pathogen avoidance, a finding consistent with recent work by Chen et al. (2024).

However, Study 1 yielded an intriguing and theoretically coherent finding: while not influencing reactions to unfairness, higher trait pathogen avoidance did predict a greater acceptance of fair offers (an effect not replicated at the state level in Study 2). Individuals with a stronger preference for spicy food reported higher trait pathogen avoidance (e.g., Chen et al. 2024; Prokop and Fančovičová 2011; Spence 2018), which in turn was associated with a lower rejection rate for equitable 50:50 splits. This result aligns with behavioral immune system theory, which posits that adherence to social norms—including fairness—can serve as a broad, prophylactic strategy to manage threats and maintain social stability (Ackerman et al. 2018; Brenner and Inbar 2015; Hlay et al. 2021; Schwambergová et al. 2023). By readily accepting fair and predictable social arrangements, individuals may reduce conflict and the associated risks, an adaptive behavior for those dispositionally high in disease avoidance.

The inconsistency in findings regarding pathogen avoidance across the two studies can likely be explained by the nature of the state‐level measure used. Indeed, this dissociation between a trait‐level association and a state‐level null effect precisely replicates the pattern reported by Chen et al. (2024). They, too, found a link between spicy preference and trait pathogen avoidance (using the PVD) but no immediate effect of consumption on situational pathogen avoidance (using the identical C‐DIS measure). The nature of this specific measure offers a direct explanation: its images are designed to be highly salient and potent elicitors of pathogen disgust (Culpepper et al. 2018). It is plausible that this powerful stimulus induced a strong disgust response across all participants, and this potent, stimulus‐driven activation may have overshadowed or masked the more subtle, transient influences stemming from spicy food consumption. Thus, the null finding in the experimental context of Study 2 does not necessarily contradict the trait‐level association observed in Study 1 (for fair offers), but rather highlights a methodological consideration regarding the intensity of the measurement tool. Future research could provide a more sensitive test of the state‐level pathway by employing measures that do not involve such explicit pathogen cues (e.g., Makhanova et al. 2021).

### 4.3 Limitations and Future Directions

Despite its contributions, the present research has several limitations that offer valuable avenues for future inquiry. First, our reliance on a university student sample from Hunan, a region of China known for its spicy cuisine, raises considerations of generalizability. However, this homogenous sample was strategically advantageous for an initial investigation. The primary aim of this research was to provide a rigorous first test of the theoretical mechanisms linking gustatory experience to fairness. By minimizing demographic and cultural variability, we could achieve a clearer assessment of the proposed psychological pathways, establishing a crucial foundation that future work can build upon. To test the robustness of these findings, future research should recruit participants from diverse cultural and culinary backgrounds, which may reveal important moderation effects related to cultural norms around food and social interaction (Ludy and Mattes 2012; Murray and Schaller 2016; Prokop and Fančovičová 2011).

Second, a methodological consideration in Study 2 is that the mediating mechanism was tested via statistical mediation. While this approach tests a theoretical model rather than offering definitive causal proof for the mediator (Hayes and Preacher 2014), our interpretation is grounded in strong empirical precedent. Our experiment established the causal links from spicy food consumption to an altered sense of fairness (the X → Y path) and to state aggression (the X → M path). The final link in the chain—the causal influence of aggression on sense of fairness (the M → Y path)—is robustly supported by an extensive body of prior research (e.g., Evans et al. 2024; Rubio‐Garay et al. 2016; Seip et al. 2014; Zheng et al. 2017). To more definitively establish the causal role of aggression in this specific context, future experiments could employ a causal mediation design, for instance, by experimentally manipulating both spicy food consumption and state aggression.

Third, our measures of spicy food preference and pathogen avoidance have limitations. The use of a single‐item self‐report to measure spicy food preference in Study 1, while common in the literature (e.g., Bègue et al. 2015; Chen et al. 2024; Ji et al. 2013; Prokop and Fančovičová 2011), may not fully capture the construct's complexity. Future research could incorporate more objective, behavioral measures, such as quantifying the amount of hot sauce participants voluntarily add to a neutral food item, to increase reliability (e.g., Bègue et al. 2015). Regarding the PVD scale, while the Cronbach's *α* for the total score in our sample was slightly below the conventional threshold, it is important to note this instrument is widely validated and utilized in behavioral immune system research (e.g., Duncan et al. 2009; Wu and Chang 2012; Wu et al. 2015). As we reported, Cronbach's *α* underestimates reliability (e.g., Padilla and Divers 2016; Wen and Ye 2011), and the scale's composite reliability in our study exceeded the acceptable threshold, supporting the use of the total score. That being said, to build a more comprehensive picture and demonstrate convergent validity, future research could fruitfully complement the PVD with other related trait measures, such as disgust sensitivity (Ackerman et al. 2021; Batres and Perrett 2020; Liuzza et al. 2019; Wu et al. 2022).

Fourth, our investigation focused exclusively on the perspective of the responder in distributive behavior, the mindset of the proposer remains unexplored (Wu et al. 2013). Future studies should examine whether spicy food preference or consumption influences how individuals formulate offers, which would provide a more complete picture of its impact on fairness. Finally, while the UG task is a standard and powerful paradigm, it does not capture the full complexity of real‐world fairness decisions, which are embedded in rich social contexts involving relationships, reputation, and long‐term consequences (Jost and Kay 2010). Future research could employ more naturalistic scenarios to explore whether the effects of spicy food extend to broader social behaviors such as altruism, cooperation, and competition (Fehr and Schmidt 2006), thereby deepening our understanding of the profound ways in which sensory experiences shape human social life.

## 5 Conclusion

The present research demonstrates that both the preference for and consumption of spicy food enhance individuals' sensitivity to unfairness, an effect consistently observed across different levels of inequity, suggesting an elevated acceptance threshold. This heightened sense of fairness was primarily mediated by aggression. Specifically, both a chronic preference for spicy food (via trait aggression) and its immediate consumption (via state aggression) resulted in a reduced tolerance for unfair offers. In contrast, the mediating role of the behavioral immune system remains inconclusive and warrants further investigation. These findings offer novel insights into how gustatory experiences shape social decision‐making and perceptions of fairness.

## Ethics Statement

This current study was performed in line with the principles of the Declarations of Helsinki and was approved by the Research Ethics Committee of Hunan Normal University. Informed consent was obtained from all participants involved in the study.

## Conflicts of Interest

The authors declare no conflicts of interest.

## Data Availability

The data that support the findings of this study are available from the corresponding author upon reasonable request.

## Endnotes

^1^
Given that Cronbach's *α* for the total PVD score was slightly below the conventional threshold of 0.70, and that Cronbach's *α* tends to underestimate reliability, we also calculated composite reliability (e.g., Padilla and Divers 2016; Wen and Ye 2011). This composite reliability value exceeds the 0.70 threshold and indicates that the internal consistency of the total PVD score was acceptable in the present study (e.g., Padilla and Divers 2016; Wen and Ye 2011; Wu et al. 2022).
